# Supplementary material for: Identifying behavioral structure from deep variational embeddings of animal motion
Source: Commun Biol. 2022 Nov 18;5:1267. doi: 10.1038/s42003-022-04080-7 (PMC9674640; doi:10.1038/s42003-022-04080-7)
Supplement: Supplementary file 2 — Supplementary Information [file 42003_2022_4080_MOESM2_ESM.pdf]

IDENTIFYING BEHAVIORAL STRUCTURE FROM DEEP VARIATIONAL EMBEDDINGS OF ANIMAL MOTION

Kevin Luxem[1,2], **Petra Mocellin**[1,2], **Falko Fuhrmann**[1,2], **Johannes Kürsch**[1,2], **Stefan Remy**[1,2,3,4,5] **and Pavol Bauer**[1,2,5]
[1]Leibniz Institute for Neurobiology, Department of Cellular Neuroscience, Magdeburg, Germany
[2]German Center for Neurodegenerative Diseases, Magdeburg and Bonn, Germany
[3]Center for Behavioral Brain Sciences, Magdeburg, Germany
[4]German Center for Mental Health, Jena-Magdeburg-Halle, Germany
[5]These authors jointly supervised this work

September 20, 2022

Supplemental Materials

1 VAME model selection

The VAME model consists of one biRNN encoder and two biRNN decoder. A HMM is used to identify hidden states (motifs) within our embedding space, as described in the main article (also see Methods). The model was chosen after we tested four different variations of the architecture and compared the HMM against a k-Means algorithm. In Table 1, we show the different choices and validated their outcome based on our benchmark dataset.

Our architectural choices were either a standard variational autoencoder consisting of a biRNN encoder and a biRNN decoder or with an additional biRNN prediction decoder. Furthermore, we applied to both variants spectral regularization of the latent space [1] to see if this could lead to improved clusterability. We applied three metrics (Purity, NMI, Homogeneity, see Methods) to identify the best model. In both cases (k-Means or HMM), the variational autoencoder model without spectral regularization and an additional decoder had the highest scores. The model with HMM led to the best scores and hence, we chose this as the primary model in our manuscript.

Model (segmentation)	Purity	NMI	Homogeneity %
VAME single decoder (k-Means)	74.66	22.26	43.02
VAME single decoder + spectral regularization (k-Means)	76.17	22.13	43.20
VAME two decoder (k-Means)	**76.23**	**23.58**	**45.55**
VAME two decoder + spectral regularization (k-Means)	75.56	23.12	44.69
VAME single decoder (HMM)	78.44	26.70	49.82
VAME single decoder + spectral regularization (HMM)	79.81	26.98	51.98
VAME two Decoder (HMM)	**80.66**	**28.61**	**54.85**
VAME two Decoder + spectral regularization (HMM)	79.15	27.64	52.84

Table 1: VAME model selection with two different segmentation algorithm (k-Means and HMM) for $k = 50$. Reported is the mean of five repeated training and inference runs.

2 Motif description

To describe the motifs we investigated the single motif videos (Examples are given in supplemental video 1-5). Motif 14 (*Turning* community) is characterized by a rotational motion with the front paws further away from each other. Motif 31 (*Stationary* community) shows no paw movement but some nose motion. Motif 34 (*Walking* community) shows a push into a slow walk motion and as shown on the hierarchical tree Figure 2, C, it is grouped further away from the other walk motifs, which suggests that it does not belong to the classical walk cycle. Motif 42 and Motif 48 (*Unsupported Rearing* community) show a stationary standing animal inside the arena with some nose motion. Stationary here refers to almost no movement in the back paws.

Fig. 1 a shows the usage comparison for the other motifs/communities that are not significantly changed in our experiment. Part B depicts the time binned evolution of the other significantly changed unsupported rearing motif 48. To specifically find out whether behavioral differences were detectable by human observation in our experimental setting, we performed human classification, which was carried out by eleven human experts, all trained in behavioral neuroethology. This classification was based exclusively on the video recordings that were used for our VAME approach. We constructed an online questionnaire for blinded classification where each participant was allowed to watch all videos for an unlimited amount of time before making their decision (see Methods **??**). Experts with previous experience about APP/PS-1 had a slightly higher classification accuracy (50.98% $\pm$ 11.04% for experts, 42.5% $\pm$ 15.61% for non-experts). The overall classification accuracy was at chance level for all participants (46.61% $\pm$ 8.41%, Fig. 1 c).

Table 2 summarizes the statistics for the significantly changed motifs based on a multiple t-Test.

Sup. Fig. 1: Additonal information about motif statistics.

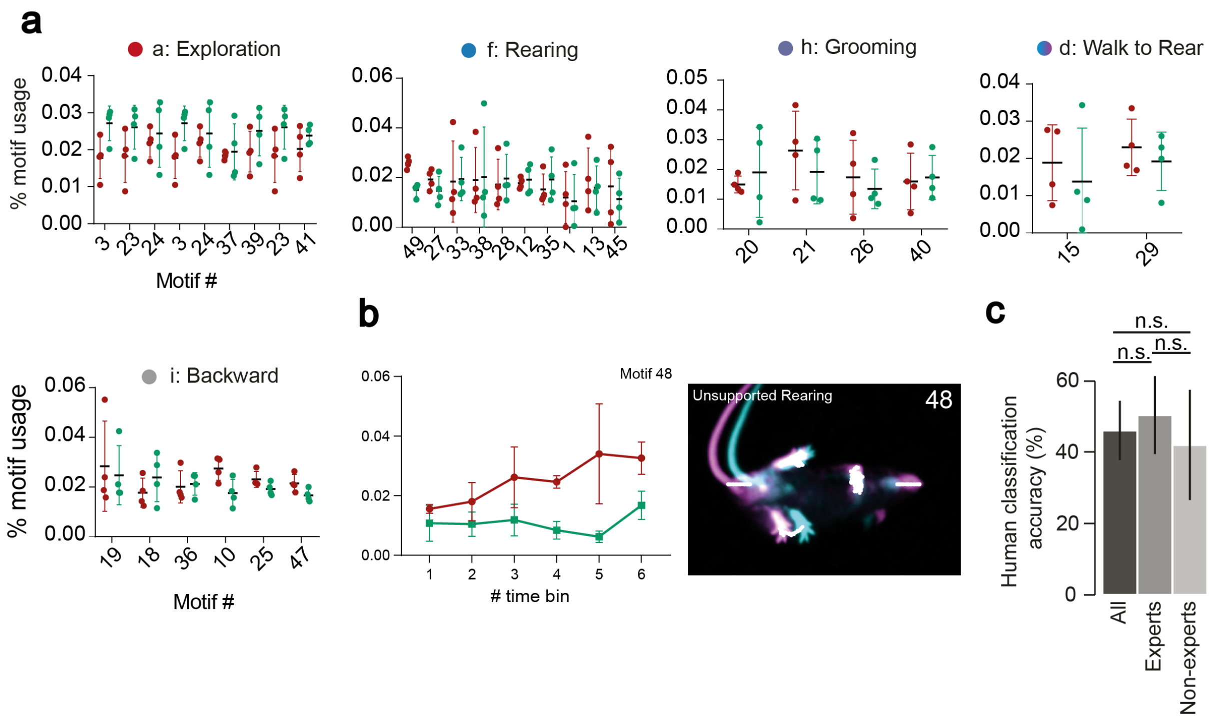

Figure 1: **a** Comparison of usage for not significantly changed motifs. **b** Time binned evolution of the significantly changed unsupported rearing motif 48 and visualization of the motif. **c** Human classification results. Error bars represent standard deviation.

Motif	Community	Mean Usage tg (%)	Mean Usage wt (%)	SE difference	T-ratio	p-Value
14	Turning	1.4	2.4 ↑	0.003	3.69	0.005
31	Stationary	2.6	3.4 ↑	0.007	2.827	0.008
34	Walking	1.4 ↑	0.9	0.004	3.839	0.0003
48	Unsupported Rearing	2.5 ↑	1.1	0.004	3.004	0.006
42	Unsupported Rearing	2.7 ↑	1.3	0.004	2.861	0.008

Table 2: Significant different motifs between tg and wt animals.

3 Motif clustering number

Deciding the number of cluster (e.g. motifs) k present in a data set is hard task when no a-priori knowledge is available. To determine this number for our dataset, we used a similar technique as presented in [2]. We took our trained VAME model and let the HMM infer 100 motifs and treated motifs that had less than 1% usage as noise. This mark was 50 motifs, which we used throughout the paper. Fig. 2 visualizes the motif usage for all eight animals (blue lines), the mean usage (red line) and the 1% threshold (dashed line). The first 10 motifs have a high usage, which then drops slowly to the 1% threshold line (48 motifs) and continues to decreae to almost 0% usage (100 motifs). Once this number has been identified, a new HMM model is trained with this number to segment the final motif distribution.

Sup. Fig. 2: Sorted motif usage for all animals.

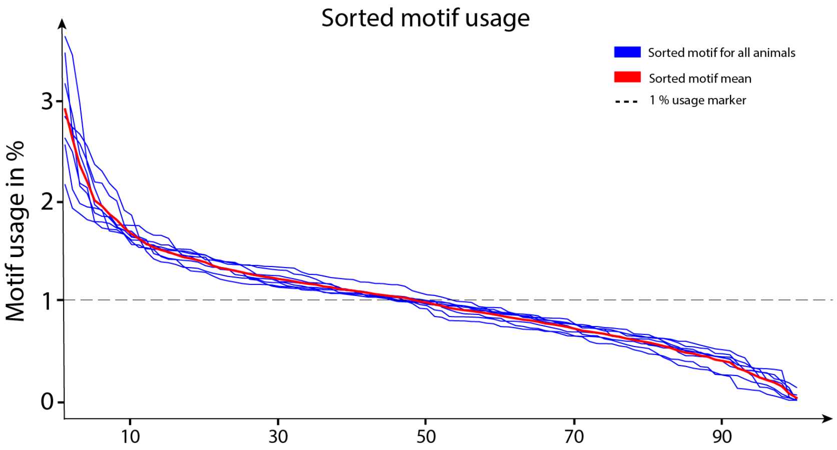

Figure 2: Sorted motif usage to identify the best number of behavioral motifs within the dataset. Blue lines representing all animals (n = 8) while the red line represents the mean usage. The dashed line represents the $1\%'$ usage threshold and is reached at about 50 motifs.

4 Parameters and biases

Although the proposed quantification method is unsupervised, the choice of parameters as well as processing steps integrate biases into the quantification result. In Table 3 we summarize the biases of our approach and state the parameter setting that have been used for our data.

Choice	Our setting
What is the pixel resolution of the animal body in the camera image	In our case, the animal body was covered with approximately 100×300 pixels.
What is the temporal resolution of the camera?	The employed camera operates at 60 Hz.
How many virtual markers are used and how are they placed on the animal body parts?	We placed 6 virtual markers on paws, the nose and the tail root (Fig. **??**).
Which software is used for keypoint detection?	We used DeepLabCut 1.1 [3].
How many frames are manually labeled?	We labeled the keypoints in 600 uniformly chosen frames.
How long is the keypoint detection CNN trained?	We trained the keypoint detection for 350.000 iterations, the resulting training errors was 2.14 pixels and the test error was 2.51 pixels.
How is the obtained keypoint time series aligned to egocentric coordinates?	We use the procedure defined in the Methods section.
How is missing data handled?	Missing data points for periods where pose estimation could not reliably detect the position of the corresponding virtual marker shorter then 200 ms were linearly interpolated while the values for longer periods were set to an arbitrary negative value.
Which size of the time window was used for the reconstruction and prediction decoder?	We used 30 frames (500 ms) for the reconstruction and 10 frames (166 ms) for the prediction decoder.
Which other hyperarameters have been used to train VAME?	All other hyperparameter settings can be found in the default configuration file available at the project website.
How many datapoints are used to train VAME?	We trained our model with 1.3 million data points.
How is the clustering carried out?	We used a Hidden Markov Model for state detection as described in the Methods section.

Table 3: Table of choices for our approach and settings for our model.

5 Community visualization and description

In Fig. 3 we visualized all nine communities by taking the start (cyan color) and end (magenta color) frame for a random community episode. White dots are representing DeepLabCut marker. Next to the visual representation, the DeepLabCut trace for this episode is shown. Community a contains motifs with exploration characteristics such as slow walking and a lot of nose movement which could be interpreted as sniffing. Community b shows mainly events in which motifs express rotational behavior. In c, the motifs display almost no movement of any body part. Community d consists of two motifs which depict transitional behavior from walk to rear or vice versa. In community e, we found that all motifs express a specific part of the walking behavior. Community f contains motifs which are mainly showing rears along the wall of the arena while g contains motifs depicting rears within the arena. Community h belongs to the same branch as g but portrays mainly motifs with grooming activity. Lastly, community i shows motifs in which the animal performs a backward motion e.g after rearing.

Sup. Fig. 3: Visualization of Communities with their respective DLC trace.

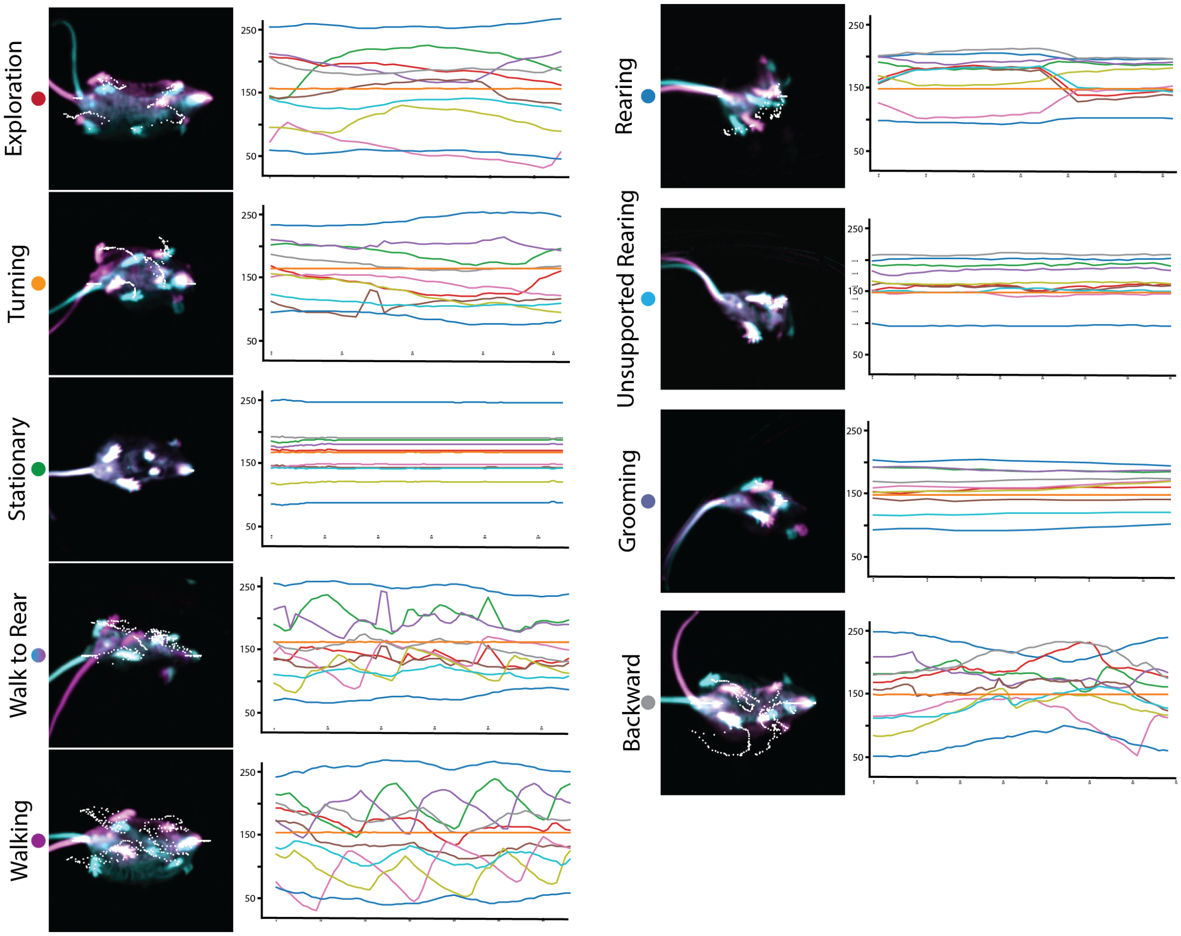

Figure 3: All nine communities that have been identified with our hierarchical tree clustering algorithm are depicted as mouse frames (temporally from cyan to magenta) with their corresponding egocentrical aligned DeepLabCut trace.

6 Generative model

The VAME framework is based on the concept of variational autoencoder, which belongs to the class of generative models. The power of a generative models lies in its ability to learn the underlying data distribution and create new, unseen samples from this distribution. This capability can be used to show that the model is able to represent the data distribution well. In Fig. 4, weshow VAME's capabilities to generate specific samples from the motif distribution as well as randomly generated trajectories.. To demonstrate that we can generate samples from VAME's latent space, we carried out ex-post density estimation using a 10-dimensional GMM, as proposed in [4]. From the fitted density, we are able to sample datapoints that can be transformed to input trajectories. With this functionality, users of VAME can verify if the model has succesfully learned the data distribution to generate realistic synthetic samples. Note that this approach can be also used to validate single clusters, if the density is fitted on regions belonging to individual motifs only. In Fig. 4 a-c, we use three exemplary motifs and sample from their latent distribution. By using the reconstruction decoder of VAME as a generative model, we can show that the distributions are well defined since the decoder is reconstructing very similar trajectory samples. Note that we did not use any input sample to decode the motif trajectories and this is all coming from the learned latent distribution. Fig. 4 d shows trajectory samples form randomly sampled data points of our latent distribution.

Sup. Fig. 4: Generative capabilities of VAME by sampling from the latent distribution

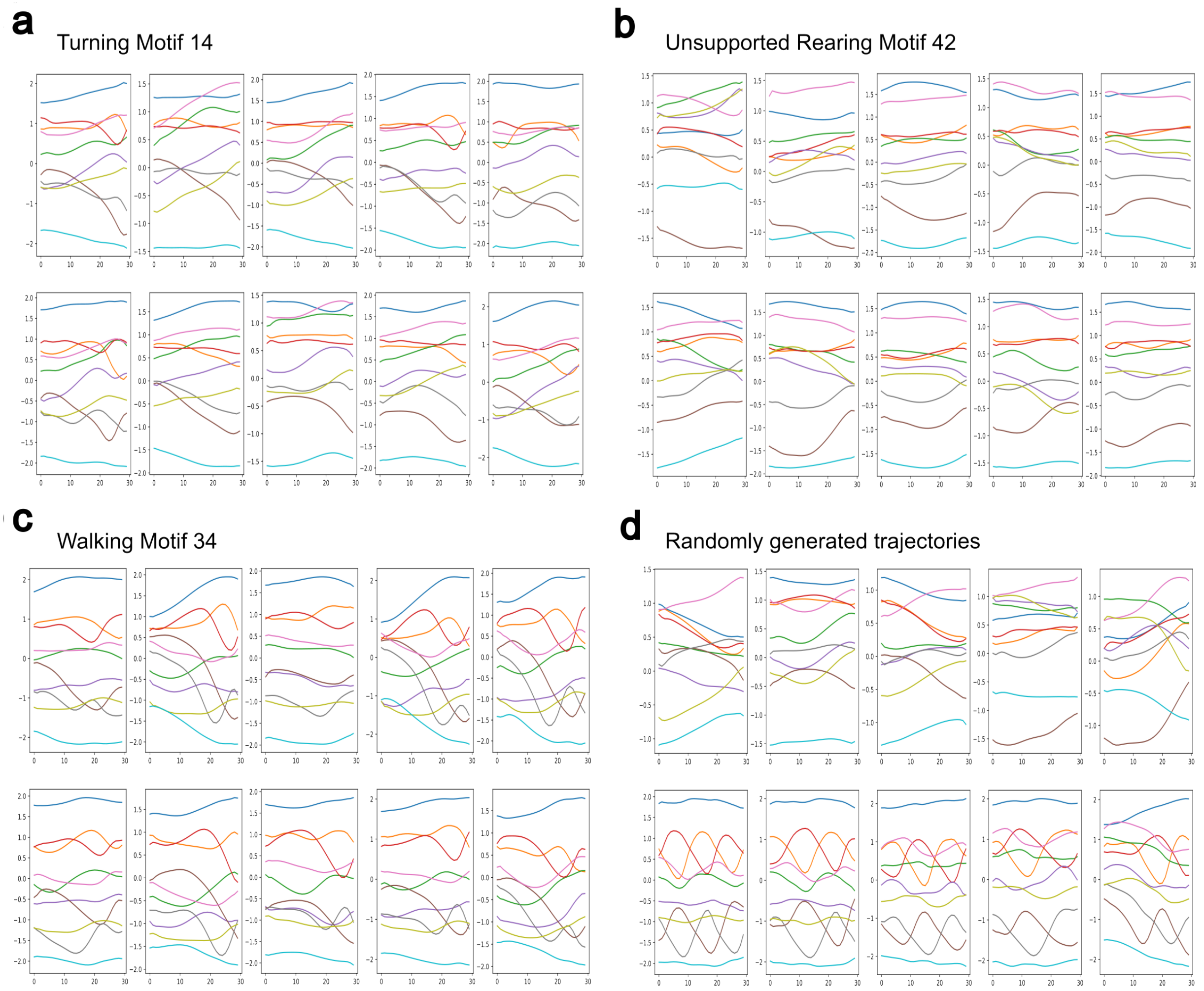

Figure 4: **a** Random generative samples of the *Turning* motif 14. **b** Random generative samples of the *Unsupported Rearing* motif 42. **c** Random generative samples of the *Walking* motif 34. **d** Generated samples obtained by fitting a Gaussian mixture model on the latent space.

7 Sequence detection in locomotion

Given the results shown in Fig. 3 c, we used the representation learned by VAME to identify locomotion patterns within the *Walking* community. Briefly, we built an algorithm that first identified reoccurring motif patterns in the community and then counts their occurrences. We detected 22 sub-patterns, which were used by all animals. Within these sub-patterns, we were able to identify four specific sub-second sequences that were significantly more used in either wt or tg mice (unpaired t-Test). The strongest sub-pattern for wt animals is the sequence [16, 32] and [32, 22, 7], which can be also seen in the difference graph as a strong transition. For the tg, the strongest pattern involves the sequence [2, 9], which again can be seen in the difference graph as a strong transition for the tg group. These results demonstrate the capabaility of VAME to robustly capture patterns for transitions between motifs, especially for locomotion behavior based on its phase sensitivity (Fig. 5).

Sup. Fig. 5: Identfication of the locomotion structure in both groups and visualization of four significant different patterns.

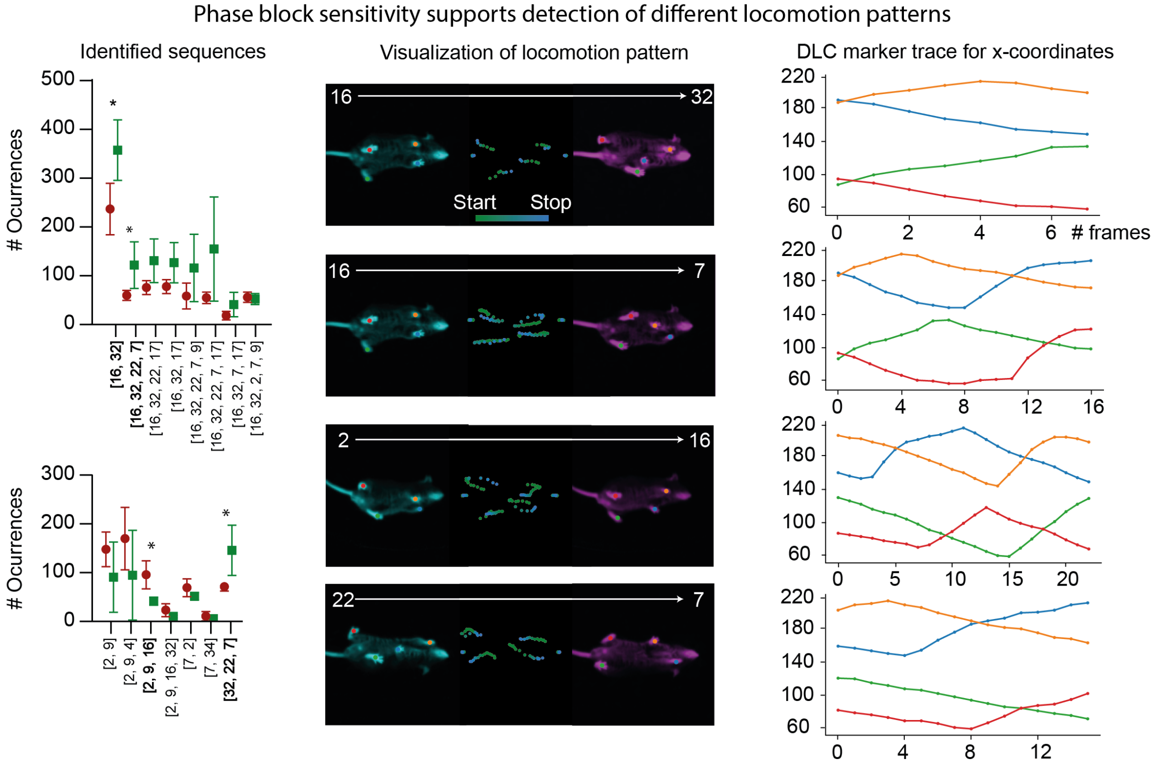

Figure 5: Error bars represent standard deviation.

8 Model comparison

For the comparison of our model to the AR-HMM we employed the original codebase supplied by the authors [2]. We used default parameter settings for all values (γ = 999, N_{lags} = 3, ν = 4), while the maximum number of states was set to the corresponding cluster size k. The sticky parameter setting was employed and the value of κ has been set to the number of datapoints, as suggested by the authors (According to the usage documentation available within the MoSeq repository).

To compare our model to the MotionMapper framework [5], we used the original codebase provided by the authors on GitHub. Here, we first converted the input signal to the time-frequency domain using the Wavelet transform (15 frequency bins in the range between 0 and 30 Hz). From the stacked spectrogram we then obtained a two dimensional t-SNE embedding (perplexity=32, learning rate=200, 3000 iterations). The watershed segmentation of the embedding space was adjusted to match different numbers of k clusters.

Fig. 6 shows a qualitative comparison between VAME and current methods (MotionMapper and AR-HMM, [5, 2]). We trained all models on our data and carried out segmentation into approximately the same amount of behavioral motifs (VAME, AR-HMM n=50, MotionMapper n=51). The exemplary trace is aligned to the motif sequences and shows two walking and rearing episodes. As reference, we selected the x-coordinate of the left hind paw (orange marker) to define a phase block, indicated by the dashed lines between time step 0 and 200 (Fig. 4). Visual inspection between the methods revealed that VAME motifs match the phase of the signal more precisely. The motif sequence obtained from AR-HMM exhibits more rapid state switches while MotionMapper has more longer lasting motifs.

Table 5 reports the results on Purity, NMI and Homogeneity for smaller cluster sizes of $k = \{10, 30\}$ for all four models (VAME, MotionMapper, AR-HMM, HMM) on our benchmark dataset.

To further compare VAME, AR-HMM and MotionMapper, we tested their ability to separate phenotypes based on their motif distribution. Here, we used the same approach as in [6]. Briefly, the authors used a linear discriminant analysis (LDA) classifier to demonstrate that differences in motif usage can be used to accurately distinguish larval zebrafish

Sup. Fig. 6: Qualitative comparison with MotionMapper and AR-HMM.

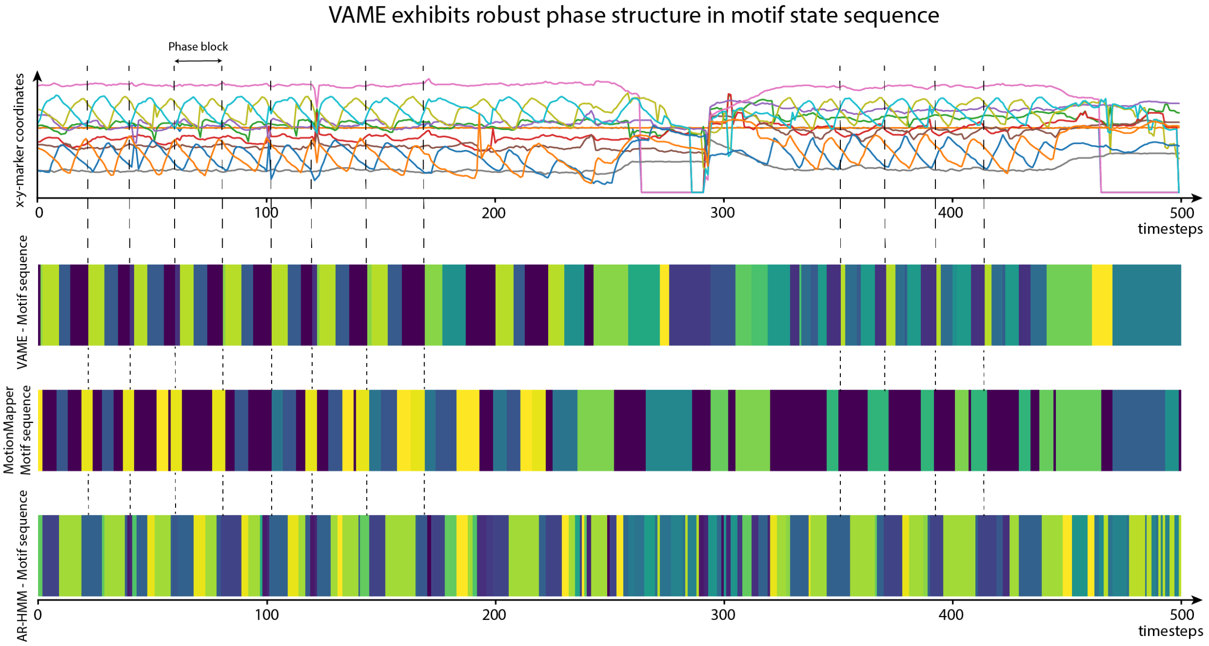

Figure 6: The figure depicts an exemplary trace of the input time series together with the motif segmentations obtained from the VAME, MotionMapper and AR-HMM methods.

k = 50	Abs. Purity	Abs. NMI	Homogeneity %
HMM	71.42	16.51	33.91
MotionMapper	70.67	17.35	30.82
AR-HMM	74.72	22.42	42.5
VAME	**80.65**	**28.61**	**54.89**

Table 4: Quantitative model comparison based on an annotated benchmark dataset.

Model	Purity	NMI	Homogeneity %
HMM ($k = 10$)	69.59	17.54	25.65
MotionMapper ($k = 10$)	62.14	10.91	15.14
AR-HMM ($k = 10$)	68.12	18.69	26.15
VAME ($k = 10$)	**74.20**	**29.65**	**42.00**
HMM ($k = 30$)	70.95	17.47	32.83
MotionMapper ($k = 30$)	66.37	14.13	22.98
AR-HMM ($k = 30$)	73.99	21.77	37.97
VAME ($k = 30$)	**79.19**	**26.10**	**51.16**

Table 5: Model comparison based on an annotated dataset with $k = 10$ and $k = 30$ motifs.

behavior across a variety of contexts, such as exposure to different concentrations of a compound. We applied the same classifier to the motif distribution output of the models tested herein. The results show that all three models can separate the phenotype to a certain degree meaning that each model has an animal misclassified. One obvious reason for this could be the strong homogeneity in behavior between the two groups. Supplemental Fig. 7 shows the LDA classification probability for each model.

Sup. Fig. 7: Linear discriminant analysis classification probabilities.

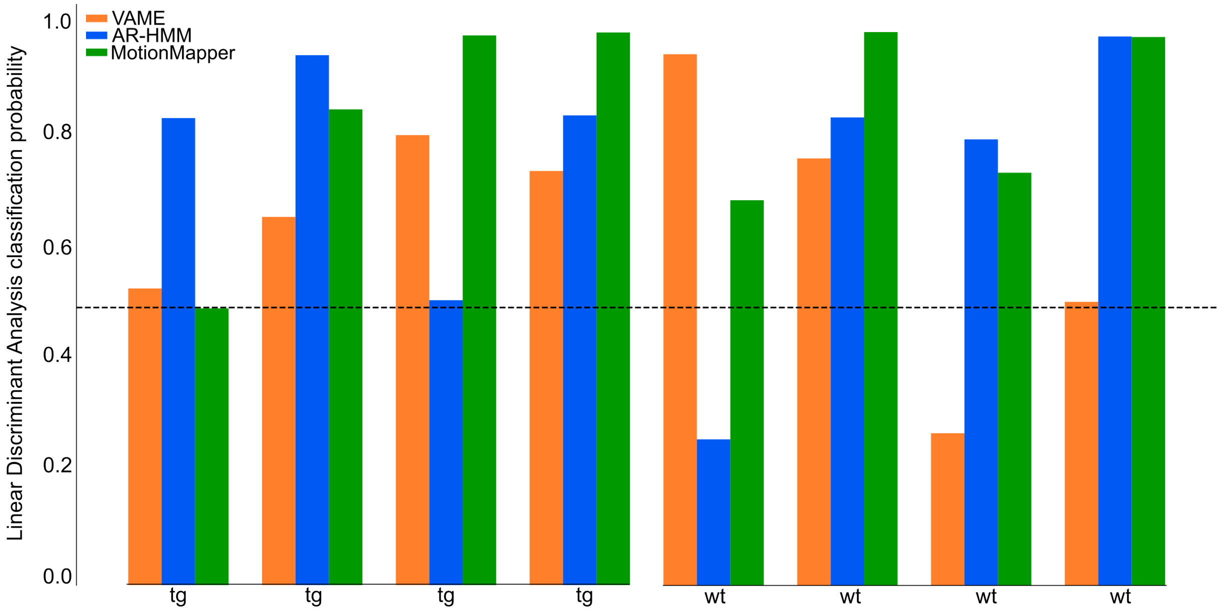

Figure 7: The dotted line indicates the 50% boundary. A probability above 50% can be seen as a correct classification while a lower probability is interpreted as a misclassification.

9 Latent projections and trajectories

In Fig. 8 a we show the Unifold Manifold Approximation (UMAP) projection of the original egocentrically aligned virtual marker signal (baseline) for the manual annotated dataset and (middle, right) the UMAP projection of the latent vectors obtained by VAME for the same dataset (middle: human label, right: VAME motifs). It can be observed that the VAME embedding is less scattered and more densely represented.

To test the spatiotemporal connectivity of the latent representation for VAME, we sampled three random behavioral sequences with a length of 1.5 seconds and plotted their trajectories on top of both visualizations. Moreover, we also plotted this trajectory on top of the t-SNE embedding of MotionMapper as comparison. We observed that the course of trajectories within the embedding of VAME followed a smooth path through the projected manifold while trajectories through the embedding of the baseline signal appears scattered. For MotionMapper, we also observed more scatter (Fig. 8 b).

Lastly, we investigated the embedding of MotionMapper more closely to understand the model behavior on our dataset (Fig. 8 c). More specifically, we show the effect of modifying the standard deviation of the smoothing Gaussian of the 2D kernel density estimation, a free parameter of MotionMapper, on the obtained t-SNE embedding as well as on obtained the watershed segmentations. As depicted here, the choice of the parameter has a strong impact on the clusterability; a high setting of the standard deviation implies a lower number of clusters that are detectable by the watershed segmentation, and vice-versa. Here, we observe that the overrepresented density in the middle of the embedding exists for segmentations to 5, 17 and 31 clusters, and is only resolved for a large setting of the cluster size (k=78). This suggests that the largest mass of the behavioral space is mostly contained in a single motif located in the center of the space. As discussed previously, this finding is likely observable for pose estimation data obtained from mice, as the sinusoidal signals have a higher similarity in the frequency space, when compared to behavioral signals measured for, for example, fruit flies.

Sup. Fig. 8: VAME latent space 2D projection and trajectories.

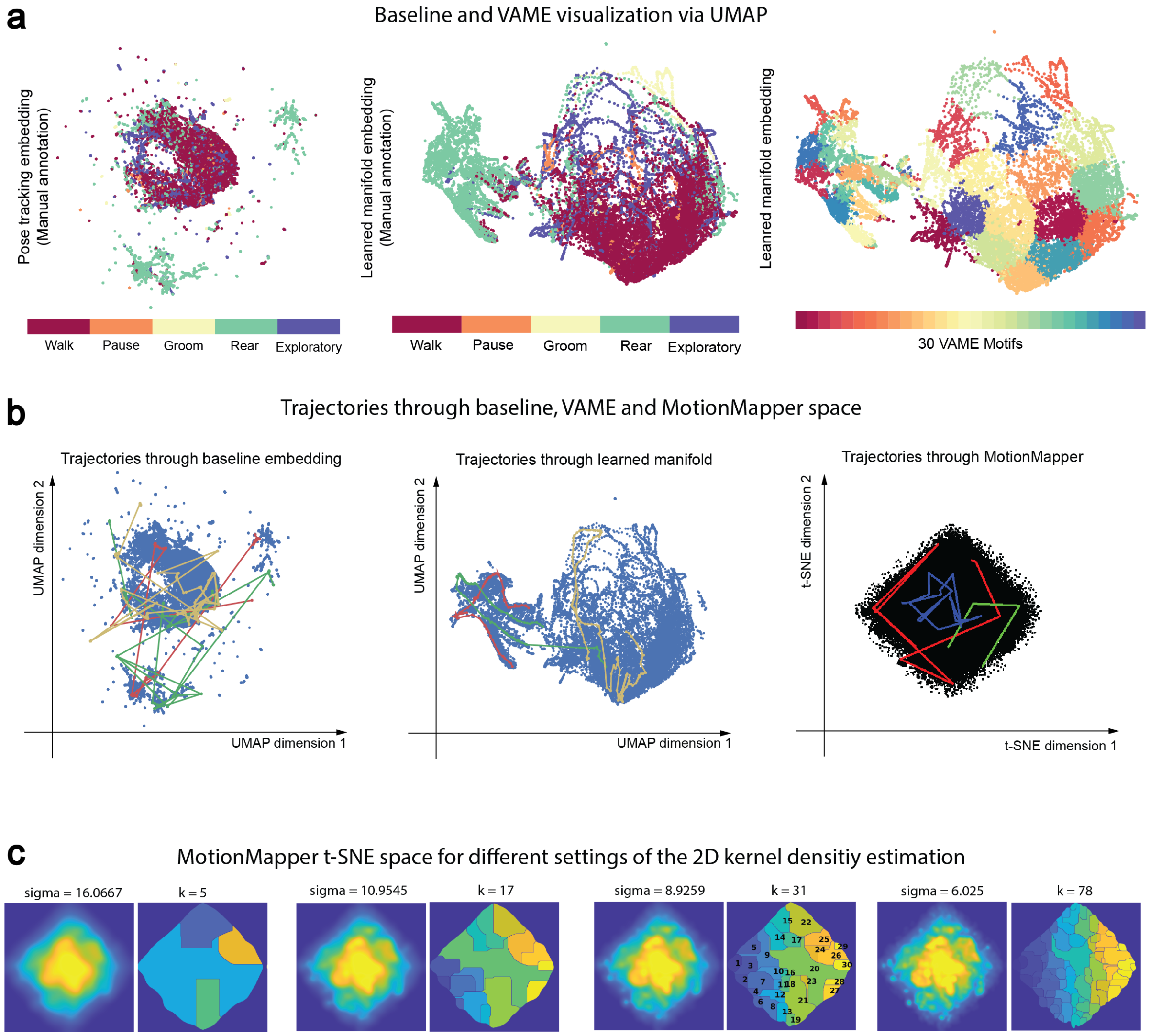

Figure 8: **a** Unifold Manifold Approximation (UMAP) embeddings of the marker position input series, the latent representation encoded from the RNN color-coded for 5 manually labeled behavioral classes and color-coded according to assignment into 30 VAME motifs. **b** Three exemplary paths of consecutive video frames through the UMAP embedding space of the spatial input series (DLC time series), the VAME embedding space showing the smooth spatiotemporal representation and t-SNE embedding obtained by MotionMapper with three examplary paths. **c** Embeddings and segmentations obtained from MotionMapper for different settings of the 2D kernel density estimation parameter (sigma).

10 Community transitions

Sup. Fig. 9: Transitions of motif within the *Stationary* and *Walking* community.

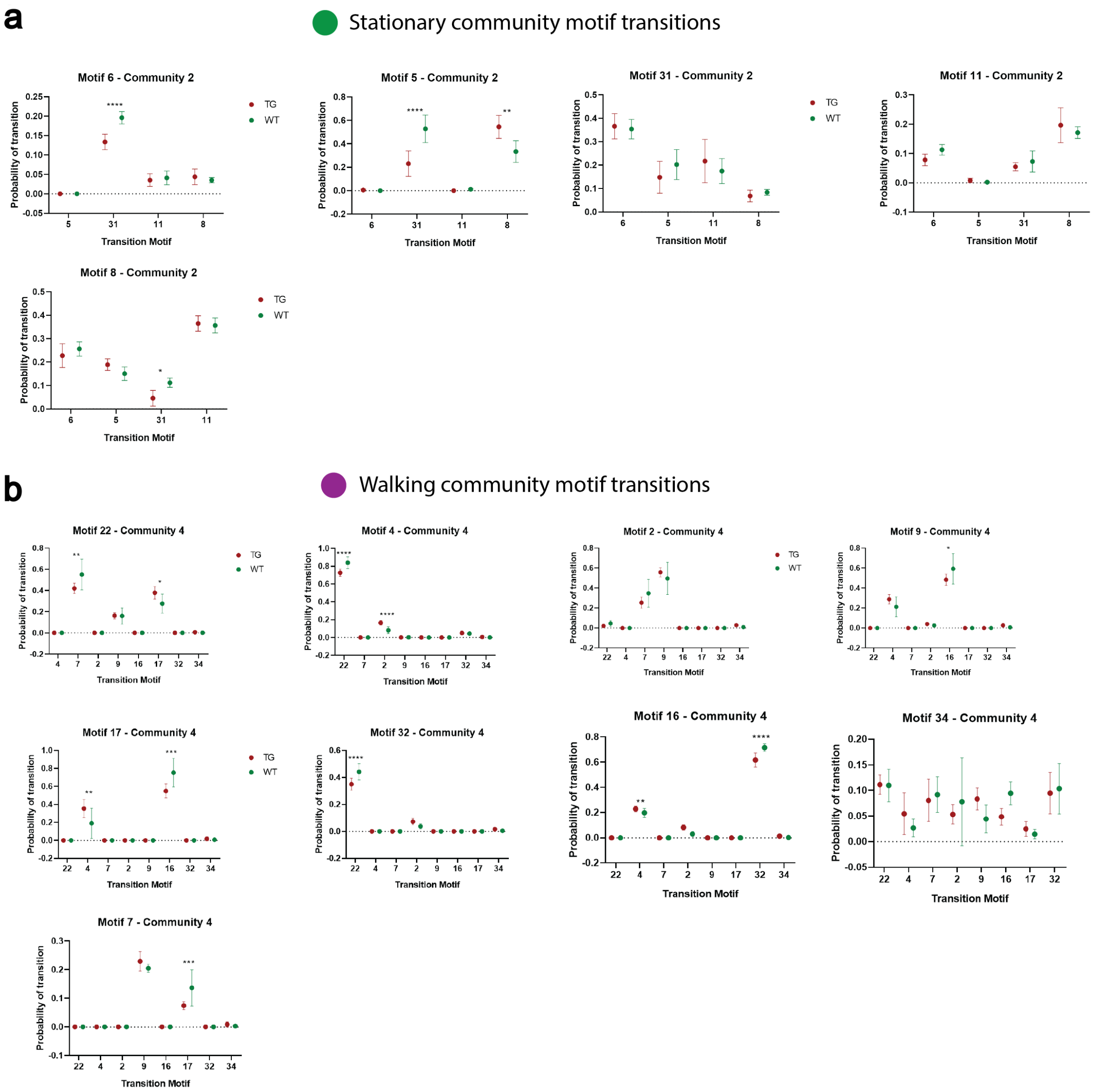

Figure 9: Error bars represent standard deviation.

Sup. Fig. 10: Within community transition statistics.

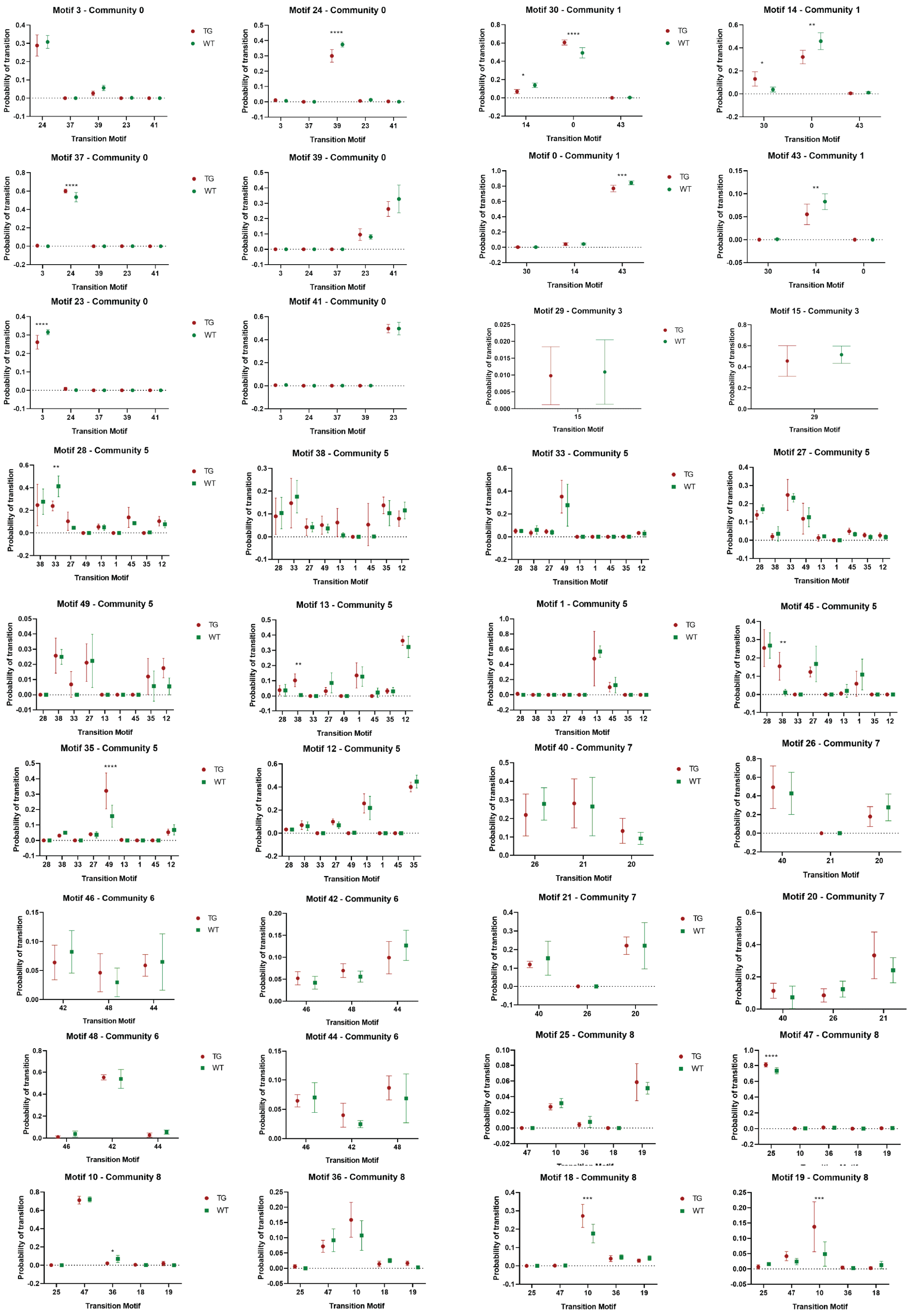

Figure 10: Error bars represent standard deviation.

Sup. Fig. 11: Example trajectories and preprocessing adjustments.

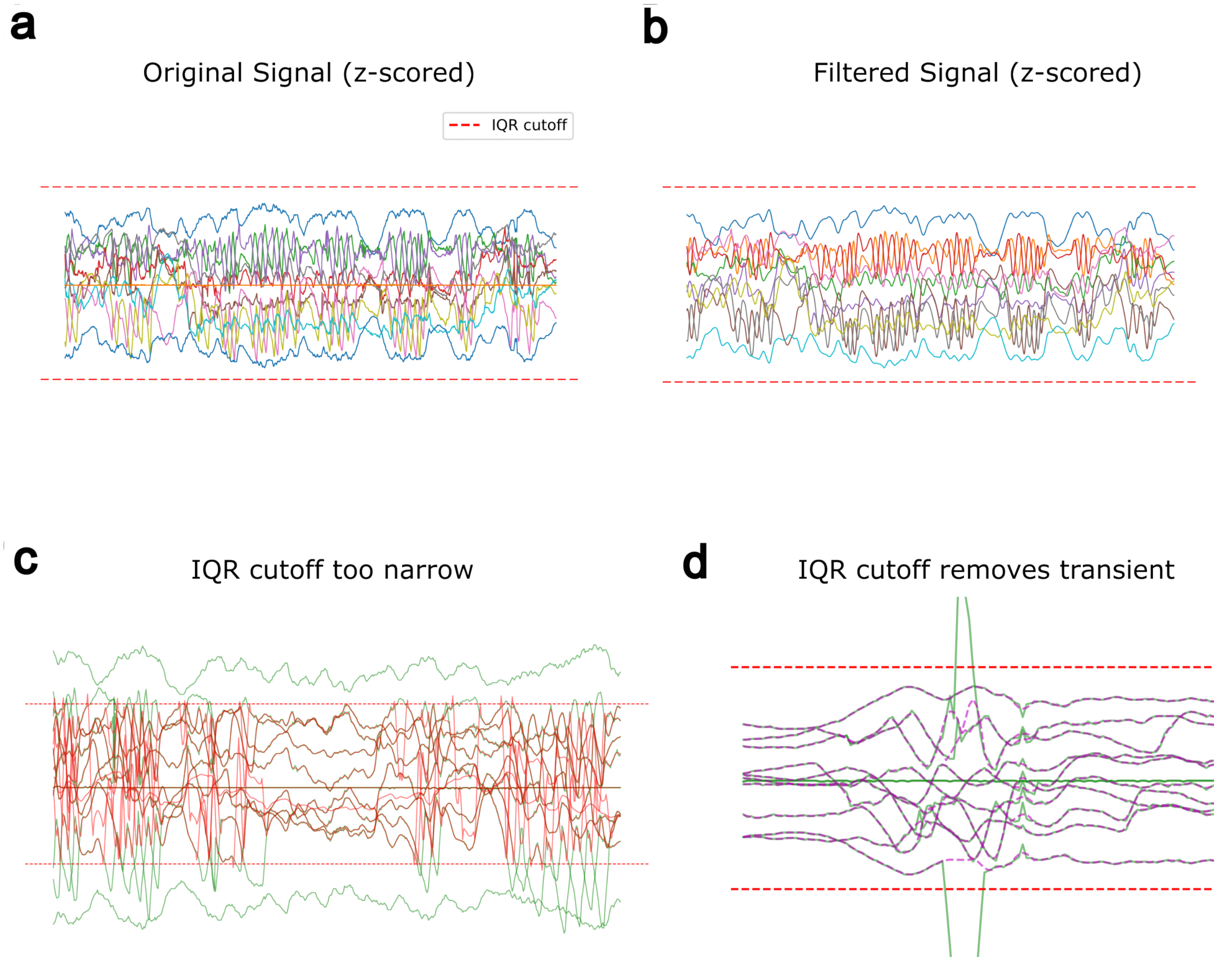

Figure 11: **a** Original z-scored signal with IQR-cutoff (red dashed line) within signal range. **b** Filtered signal (z-scored) where middle line is removed (anchor point from egocentrical alignment). **c** Example of a too narrow chosen IQR-cutoff vlaue. Here, the behavior signal (green) is cut and information is lost. **d** Example of removing a transient event in the data (green signal) with the IQR-cutoff and interpolating the signal (purple).

Sup. Fig. 12: Reconstruction and prediction examples of the input trajectories for VAME.

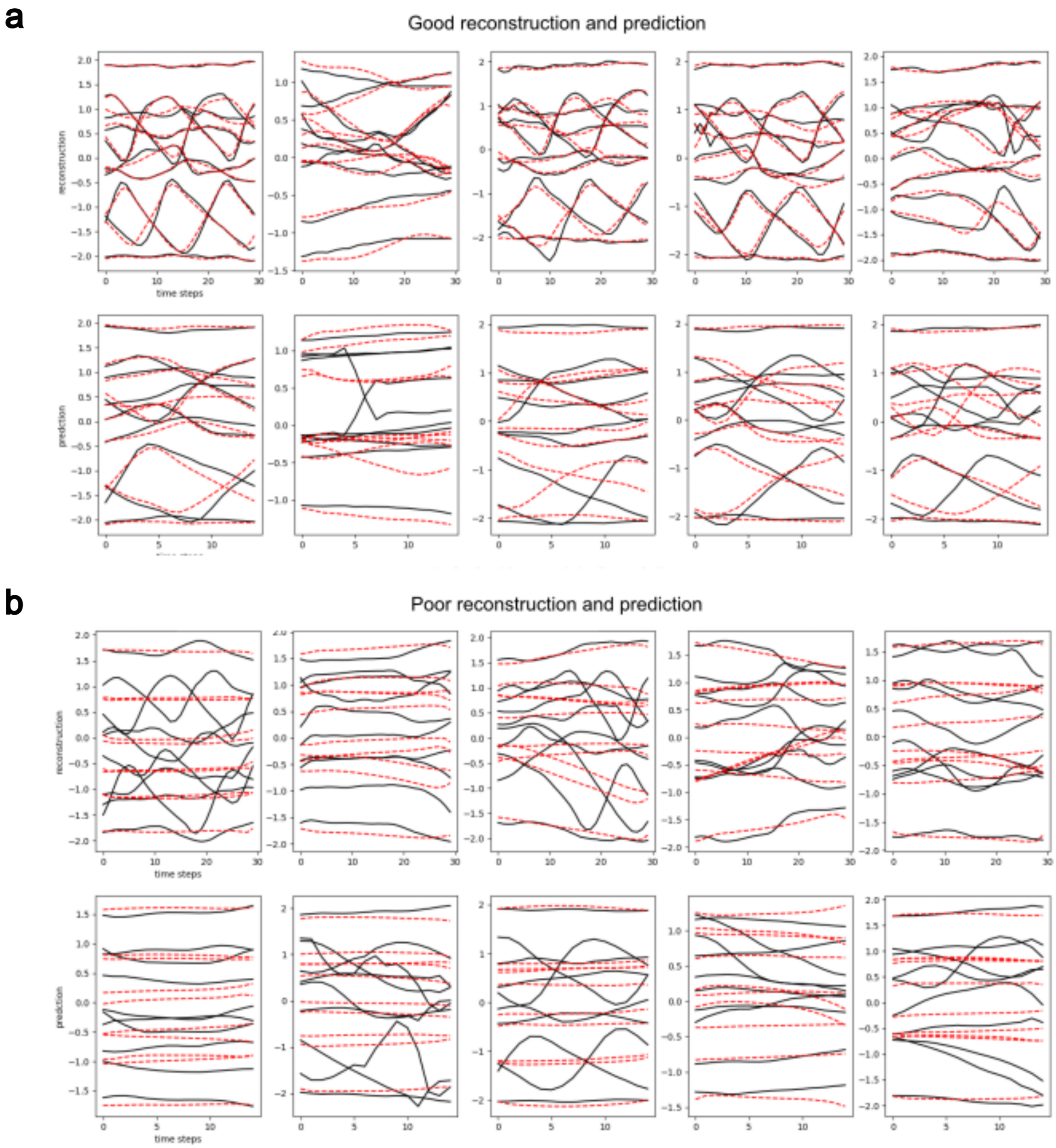

Figure 12: **a** Good qualitiy reconstruction and predictions. **b** Poor qualitiy reconstruction and predictions.

Sup. Fig. 13: Identification of the number of latent dimension z.

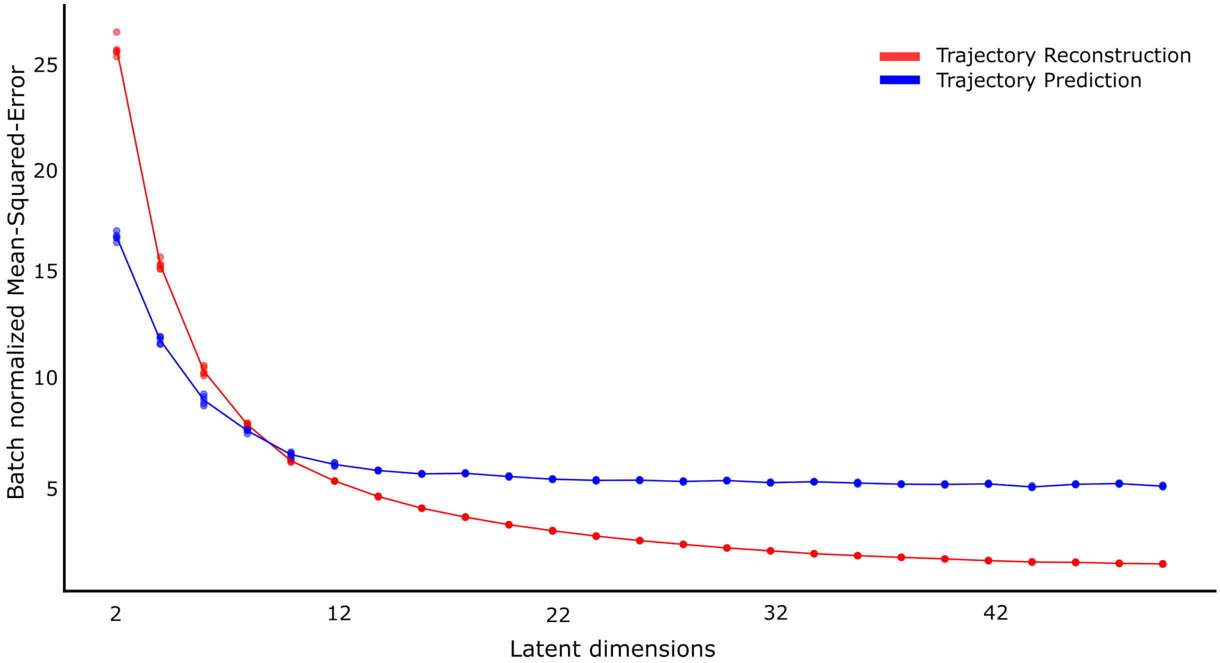

Figure 13: For a given dataset (here demonstration data used in Methods) the batch normalized mean-squared-error for the reconstruction and prediction capabilities of the VAME network can be measured for different values of z.

References

[1] Qianli Ma, Jiawei Zheng, Sen Li, and Gary W Cottrell. Learning representations for time series clustering. In *Advances in Neural Information Processing Systems 32*, pages 3776–3786, 2019.

[2] Alexander B. Wiltschko, Matthew J. Johnson, Giuliano Iurilli, Ralph E. Peterson, Jesse M. Katon, Stan L. Pashkovski, Victoria E. Abraira, Ryan P. Adams, and Sandeep Robert Datta. Mapping Sub-Second Structure in Mouse Behavior. *Neuron*, 88(6):1121–1135, 2015.

[3] Alexander Mathis, Pranav Mamidanna, Kevin M. Cury, Taiga Abe, Venkatesh N. Murthy, Mackenzie Weygandt Mathis, and Matthias Bethge. DeepLabCut: markerless pose estimation of user-defined body parts with deep learning. *Nature Neuroscience*, 21(9):1281–1289, 2018.

[4] Partha Ghosh, Mehdi S. M. Sajjadi, Antonio Vergari, Michael Black, and Scholkopf. From variational to deterministic autoencoders. In *International Conference on Learning Representations*, 2020.

[5] Gordon J. Berman, Daniel M. Choi, William Bialek, and Joshua W. Shaevitz. Mapping the stereotyped behaviour of freely moving fruit flies. *Journal of The Royal Society Interface*, 11(99):20140672, 2014.

[6] Marcus Ghosh and Jason Rihel. Hierarchical compression reveals sub-second to day-long structure in larval zebrafish behavior. *eNeuro*, 7(4), 2020.
