# Supplementary material for: Identifying behavioral structure from deep variational embeddings of animal motion
Source: Commun Biol. 2022 Nov 18;5:1267. doi: 10.1038/s42003-022-04080-7 (PMC9674640; doi:10.1038/s42003-022-04080-7)
Supplement: Supplementary file 3 — Description of Additional Supplementary Files [file 42003_2022_4080_MOESM3_ESM.pdf]

Description of Additional Supplementary Files

File name: Supplementary Movie 1

Description: A video of motif 14 detected by VAME

File name: Supplementary Movie 2

Description: : A video of motif 31 detected by VAME

File name: Supplementary Movie 3

Description: A video of motif 34 detected by VAME

File name: Supplementary Movie 4

Description: A video of motif 42 detected by VAME

File name: Supplementary Movie 5

Description: A video of motif 48 detected by VAME

File name: Supplementary Movie 6

Description: A video of community a (exploration) created by the tree clustering algorithm

File name: Supplementary Movie 7

Description: A video of community b (turning) created by the tree clustering algorithm

File name: Supplementary Movie 8

Description: A video of community c (stationary) created by the tree clustering algorithm

File name: Supplementary Movie 9

Description: A video of community d (walkto-rear) created by the tree clustering algorithm

File name: Supplementary Movie 10

Description: A video of community e (walking) created by the tree clustering algorithm

File name: Supplementary Movie 11

Description: A video of community f (rearing) created by the tree clustering algorithm

File name: Supplementary Movie 12

Description: A video of community g (unsupported rearing) created by the tree clustering algorithm

File name: Supplementary Movie 13

Description: : A video of community h (grooming) created by the tree clustering algorithm

File name: Supplementary Movie 14

Description: A video of community i (backward) created by the tree clustering algorithm
